# Unmasking Reflexivity in HR Managers During the COVID-19 Lockdown in Italy

**DOI:** 10.3389/fpsyg.2020.588128

**Published:** 2020-12-23

**Authors:** Silvio Carlo Ripamonti, Laura Galuppo, Giulia Provasoli, Angelo Benozzo

**Affiliations:** ^1^Department of Psychology, Catholic University of Milan, Milan, Italy; ^2^Department of Human and Social Science, University of Valle d’Aosta, Aosta, Italy

**Keywords:** organizational change, crisis management, HR managers’ reflexivity, qualitative approach, thematic analysis

## Abstract

This paper explores how some Italian HR managers narrate the changes imposed by the COVID-19 threat in the workplace. Events since December 2019 have presented exceptional circumstances to which HR managers have reacted in very different ways. This study explored how HR managers came to introduce organizational changes aimed at coping with the emergency, as well as how employees were involved in those organizational changes. The article is based on a thematic analysis of some interviews with Italian HR managers whose companies decided to switch working from home on a massive scale. We wanted to offer some reflections on the actions taken by a few HR managers and Italian companies to keep working at a time when most workers were forced to respect the lockdown.

## Introduction

Over the weekend before Monday 10 March 2020, Italian society, its companies, and economy stopped almost entirely. Offices, factories, and shops were closed. Traveling around the country by car or train was forbidden, and street police checks and heavy fines were introduced for those who did not respect the rules. During that period of quarantine, from the beginning of March to the beginning of May, many changes took place in the workplace. Which of these changes will remain, and which will be abandoned? In this situation, what happened to managers, workers, and jobs?

When the lockdown quarantine began, the glittering white-collar office towers designed by architects fell empty and a large-scale unplanned experiment began. Politicians, the media, and workers called it “smart working.” To be precise, however, this was not “smart working”^1^. What was witnessed and experienced was working from home or working at a distance. In those months, millions of Italian workers stayed away from their workplace. The pandemic made it necessary to organize numerous activities, requiring interpersonal collaboration through video-based business meetings. Facebook, Microsoft, and Google decided to resort to remote working on a massive scale, until after the summer. Some firms went even further and for example, at the social media company, Twitter employees were allowed to work remotely indefinitely. Working from home was one of the many possible ways to react to the emergency, which involved a transition for millions of workers who had never experienced this way of working before.

This study aims to explore how some Italian HR managers narrate the changes imposed on the workplace by the restrictions of the COVID-19 pandemic. What has happened in the world since December 2019 has been an exceptional event to which HR managers have reacted in very different ways. We were interested in understanding how HR managers came to introduce organizational changes aimed at coping with the emergency, as well as how employees were involved in those organizational changes. The article is based on a thematic analysis of some interviews with Italian HR managers whose companies decided to resort to working from home on a massive scale. We wanted to offer some reflections on the actions taken by a few HR managers and Italian companies to keep working at a time when most of the workers were forced to respect the lockdown.

## Theoretical Framework

### The Crisis Management

This paper draws on theories of crisis management to understand how companies and HR managers deal with exceptional situations/events. According to Giust-Desprairies (2005), a crisis presents itself as an unexpected event, upsetting the *status quo*. From a psycho-sociological perspective, several aspects characterize the experience of a crisis: emotional overload; a sense of uncertainty and loss; disorientation, and disengagement. At the organizational level, crisis usually brings to the fore certain issues, needs, and expectations that were previously overlooked; the framework and knowledge used for orienting thoughts and actions lose significance and relevance and people feel continuously threatened. Consolidated leadership and balances of power vacillate and conflicts and divergent opinions may suddenly emerge. Coordination systems get jammed. Crisis confuses people, and decision-making can be blocked, or instead dominated by reactive and impulsive stances. People often ask themselves what makes sense. The individual and collective imaginary seem to “freeze,” and the future appears as a never-ending present, characterized by a continuous escalation of emergency (Hsiu-Ying Kao et al., 2020; Galuppo et al., 2019). The COVID-19 emergency can be narrated by HR managers as either an irreversible crisis or as a temporary one. In the former, it is represented as a tsunami, which changes (and changes radically) ways of working, managing human resources, and imagining the development systems for evaluating people. According to this perspective, the world (and global business) will never be the same after the pandemic. In the situation in which the crisis is seen as temporary, there is a risk that the change will only be superficial, and the transformation generated by the threat is temporary: it is an ephemeral transition.

It is useful to ask how organizations and HR managers can learn from a crisis. One suggestion comes from theorists in the field of organization studies who have studied critical reflexivity. We are thinking, in particular, of the works of Cunliffe (2003), Alvesson (2003), Ripamonti and Galuppo (2016), Ripamonti et al. (2016, 2018), and Caetano (2015) who emphasize the important role and contribution of reflexivity to management and organizational learning (Cassell et al., 2019). As Cassell et al. (2019) wrote, the literature on this topic outlines a variety of benefits that come from reflexivity, including “enabling us to think about our thinking. and in questioning our own taken-for-granted beliefs and those of others” (p. 3). Managers and employees can learn to cope with extraordinary situations - crisis or emergency situations – that involve stopping and/or slowing down (Stewart, 2007) without making a rapid assessment and taking action, without discarding what is not entirely comprehensible or fully understood (Benozzo and Gherardi, 2019). Although difficult, situations that are anxiety-provoking and confusing can be fruitful and enriching.

The themes evoked by the COVID-19 crisis are linked to literature on crisis management, and this literature has pointed out two important issues which organizations face:

• *Influencing external stakeholders*. This issue relates to how external communication with stakeholders is managed, including which messages to send them to indicate the organization’s ability to manage the unexpected crisis event. Usually, in the context of a crisis, great attention is paid to how stakeholders react to a critical event and to how information about the organization involved is managed.
• *Internal HR management dynamics*. This issue focuses on how the organization manages three specific moments: the pre-crisis, crisis management, and the post-crisis event. The possibility of learning from this crisis management process depends on the cognitive and emotional skills and abilities of the managers involved (Pearson and Mitroff, 1993; Alpaslan et al., 2009; Williams et al., 2017).

The ability of HR management to place issues connected to taking care of people at the center of the business strategy makes it possible to implement adequate, suitable responses to environmental requirements (Bader et al., 2019). Taking care of employees is also connected to how HR management influences sensemaking processes (Weick, 1999; Velasco et al., 2013). Some authors (Hewett et al., 2017) have argued that there can be significant differences in perception of the same event between the various groups of company population. Often, the way that a critical event is interpreted can be different between managers and employees and generate situations of conflict.

In literature on crisis management, one of the central themes is internal communication. As Hewett et al. (2017) have pointed out, this means that, when dealing with critical situations, HR management must ensure that communication is transparent, clear, and authentic. This should ensure that messages regarding the aims and procedures of HR policy are correctly understood by line managers and that these are then passed on to collaborators. A positive perception of the action taken by management in the face of a problematic situation has a positive impact on organizational performance (Pombo and Gomes, 2019).

### Crisis Management in the Period of COVID-19

COVID-19 is a crisis event of exceptional nature since it has affected all organizations, thereby creating a negative impact on every single organization simultaneously. In this scenario, our focus is on the role of HR management, which the literature on crisis management has always identified as being the most significant factor in influencing the capacity of an organization to react in the face of critical events. We concentrated attention on the management actions implemented by the HR department. Our research question was aimed at understanding how HR departments gave meaning to what was happening and how they built people management strategies to cope with the crisis. The issue of the relationship with stakeholders was left in the background, since COVID-19 is an event that, for the first time, has affected every organization simultaneously and brought turbulence to the whole system, meaning that organizations were left to themselves to manage internal problems. It was thus impossible to devote time and resources to dealing with external stakeholders, who were themselves having to cope with the same dramatic problems.

### The Management of Human Resources

The issues raised by the COVID-19 situation that HR management has had to deal with can be summarized as follows:

#### Stress Management

This has become a priority in the period of COVID-19. A study published on March 23, 2020, of 1257 healthcare workers in 34 hospitals in China (Lai et al., 2020) found a high prevalence of mental health symptoms among workers. Rates of psychological suffering were high: 50.4% had symptoms of depression, 44.6% of anxiety, 34% of insomnia, and 71.5% of psychological distress. The perception of not being able to respond to the increasing demands of the job during the COVID period generates a feeling of performance anxiety (Shanafelt et al., 2020). This feeling may cause a collapse in motivation and performance when the stressors exceed people’s endurance (Vogel and Bolino, 2020).

#### Management of Internal Communication

Clear, transparent communication is the primary goal during periods of crisis. Some recent studies (Garfin et al., 2020; Rao et al., 2020) on the effect of people’s repeated exposure to communication regarding the COVID-19 pandemic suggested that ambiguous messages cause increased anxiety and stress. In the COVID-19 situation, it was impossible to transmit clear messages and unambiguous responses to questions such as: “What will happen if someone is infected? Would it be possible to work from home with the same level of protection?” (Dragging et al., 2020). However, despite this lack of information, HR managers had to adopt a position and continue to give directives to company staff.

#### Generating a Supportive Atmosphere in the Workplace During the Crisis

Hou et al. (2020) studied the mental health of healthcare workers in China during the period of COVID-19. The latest research on the global pandemic indicates that the worker’s perception of social support is a protective factor that helps to contain stress. When workers are convinced that their colleagues can help them, their resilience capacity increases (Hou et al., 2020).

All these tensions have been interpreted in different ways by different organizations. Some managers have focused on ways of reassuring people. Some of the proposals reported in the literature include meditation, yoga, and stress management courses (D’Angelo et al., 2018). These experiences might have a positive influence on how future emergencies are dealt with (Hou et al., 2020). Other organizations have devoted their energy to creating a reassuring organizational environment. Yet others have tried to insist on internal communication, sending reassuring messages to their employees. A wide variety of actions have been implemented to try to deal with the pandemic, but the choices of which actions to implement depend on the meaning which the managers (and in particular those in charge of HR) attributed to the COVID-19 pandemic as an event. The specific function attributed to HR managers is to manage relations with personnel.

#### The Reflexive Function in HR Management

The COVID-19 emergency can be narrated by HR managers as either an irreversible crisis or as a temporary one. In the former approach, it is represented as a huge and sudden change, which will radically change ways of working, managing human resources, and imagining the development systems for evaluating people. According to this perspective, the world (and global business) will never be the same as before the pandemic.

In the latter narration, when the crisis is seen as temporary, there is a risk that changes will only be apparent or superficial, the transformation generated by the threat is temporary, and that this is an ephemeral transition. Once the crisis has been overcome, the organizational structure folds back on itself, because organizations are characterized by inertia, by a lack of flexibility (Gagliardi, 1986). In this second case, the role of the HR manager is to use logical arguments to convince people and contain their anxiety. In this case, there is no deep leaning or change and a return to normal is desired. Unfortunately, and paradoxically, in this situation, this mindset is not aware that normal behaviors are exactly what generated the crisis.

It is useful to ask how organizations and HR managers can learn from a crisis. Organization studies on reflexive practice, in particular of the works of Cunliffe (2003), Alvesson (2003), Ripamonti et al. (2016), and Caetano (2015), emphasize the important role of reflexivity to management and organizational learning (Cassell et al., 2019). As Cassell et al. (2019) wrote, there is literature that outlines a variety of benefits arising from reflexivity, including “enabling us to think about our own thinking. and in questioning our own taken-for-granted beliefs and those of others” (p. 3).

We refer to reflexive practice by identifying it as the ability to generate new perspectives and insights (Alvesson et al., 2008) and create scope for re-constituting the self (Cunliffe, 2003). Recently, the concept of reflexivity has been utilized in a whole series of organization studies (Chia, 1996; Holland, 1999; Weick, 1999; Antonacopoulou and Tsoukas, 2002; Alvesson, 2003; Alvesson et al., 2008; Cunliffe, 2009; Segal, 2010; Xing and Sims, 2012) to describe that process that allows people to think about themselves in their workplace by implementing a strategy of distancing themselves from the contingent situation. The aim is to activate a kind of thinking about which assumptions are used when implementing specific organizational actions (Lewis and Kelemen, 2002; Vince, 2002; Johnson and Duberley, 2003; Schippers et al., 2008; Jordan et al., 2009).

Reflexivity can be thought of as an “internal conversation” that organizational actors use to think about events that occur and to overcome the obstacles they encounter (Archer, 2007; Kakavelakis and Edwards, 2012). Through reflection, people evaluate their social contexts, imagine possible alternatives for their actions, and work with others to make organizational actions happen. In Archer’s perspective, reflexivity is an actor’s ability to build strategies for action. Strategies that take into account the actor’s position within a social system, to be able to act through tactical and strategic thinking. In this paper, we connect the concept of reflexivity to Emirbayer and Mische’s concept of agency (1998) – the ability to influence the surrounding world based on one’s ability to interpret the peculiarities of the context in which one finds oneself – which brings a temporal perspective to the discussion.

According to Emirbayer and Mische (1998), agency can refer to three temporal orientations, in the past (iteration), future (projectivity), and present (practical evaluation). In their own words, all “three of these constitutive dimensions of human action are found at various levels, within each concrete action” (Emirbayer and Mische, 1998: 171). In any given situation, however, one of these predominates, while the other two are present in a latent state. In the case of iteration, the authors refer to the selective reactivation of past thought patterns by organizational actors. Projectivity, on the other hand, involves the reconfiguration of acquired thought patterns to deal with plans. Practical evaluation concerns people’s ability to organize action in response to problems and requirements emerging in the present (Emirbayer and Mische, 1998).

We may encounter an iterative type of reflexivity that tends to select practices or thoughts from the past to deal with emerging problems. We may also encounter a kind of reflexivity centered on the present, and aimed at understanding which implicit assumptions are being used to deal with contingent problems. We may also have a style of forward-looking reflexivity, adopted to organize future strategic action.

The type of strategies chosen by HR managers therefore depends on their reflexive capacity and priorities. Bringing into play an iterative agency mainly involves thinking about which models from the past can be used to deal with the critical situations developing today. Using an approach centered on projective agency (forward-looking agency) means recognizing that the crisis has reconfigured the organizational field of action. It, therefore, becomes necessary to completely redesign how the work and the organization are conceived to guarantee survival in the medium and long term. Implementing a “practical” style of agency means placing oneself in the present and taking actions that solve strictly topical problems. In our opinion, where one positions oneself in one of these three types of agency also depends on the type of reflexive thinking to which the protagonists on the organizational scene gain access.

The priorities for action are identified by each manager’s reflexive capacity, which might favor a way of managing that is either oriented toward the past, reorganizing the present, or one which is forward-looking and focused on creating new work scenarios. For HR managers, the potential for achieving distance from the critical situation of COVID-19 depends on their critical and reflexive capacity.

## Method

The present research aimed to explore how HR managers made sense of the COVID-19 crisis, with specific regard to people management and its related changes and challenges. The study takes the form of a brief qualitative research report, whose preliminary findings are based on 10 semi-structured interviews conducted with Italian HR managers whose companies decided to resort to large-scale smart working. These multifaceted data allowed us to gain initial insights into how HR managers from different contexts spoke about the situation and made meaning from the challenges it presented.

Given the exceptional nature of the ongoing event, in the course of our research, we met up with HR managers who were willing to undertake an interview. It was not possible to proceed with a statistical sampling that took into account variables such as age or gender of the interviewees or the size of the organization. The participants in the study work in companies in the North of Italy and were identified thanks to the researchers’ network of acquaintances and by word of mouth. This process generated a convenience sample. All the interviewees gave written consent to the recording the interview and to participating in the research. All the interviews were transcribed verbatim.

Since we do not consider the narratives used by the participants in our study as representative of the HR managers’ narratives during COVID-19 lockdown in Italy, other studies are needed in this field, such as those that take into account the opinions of managers working in different or similar sectors.

The semi-structured interviews consisted of a series of open questions, organized into five main sections: a brief overview of the organization; how the organization was dealing with the current crisis; the role of HR in the COVID-19 emergency; perceived challenges, resources and criticalities in “managing people” during the crisis; expectations and predictions about the future.

### Data Analysis

Data were analyzed using a theoretical thematic analysis (Braun and Clarke, 2006), driven by the research question. The analysis aimed to identify the main themes and meanings through which the HR managers’ narratives could be organized and interpreted. The analysis was conducted as follows: in a first phase, after becoming familiar with the data, we organized them through an open coding process, in which we were driven by the research question and by the “sensitizing concepts” described in the theoretical framework (Maguire and Delahunt, 2017). We then organized the codes into broader themes that were consistent with the research question. These themes were reviewed several times, by going back and forth to the coding process. The next section presents the themes discussed by HR managers during the interviews.

The first theme regards the time orientation of managers’ actions. Managers described their commitment to cope with the present situation and to make decisions about the future. Some of them seemed focused on the present, and on the task of defending the company and its employees from threat, by trying to limit permanent damage to the organization’s capacity for survival. From the reflections of other HR managers, the belief emerged that, due to the huge crisis, the real battle was with the future and opportunities to introduce changes that might increase the ability of the company to develop.

The second area, on the other hand, distinguished between two different ways of reacting to the emergency, one conservative and the other transformative. The former tended to emphasize the possibility of re-establishing the pre-COVID situation, and it thus becomes important to devote the maximum amount of energy to keep the organization alive and wait for the crisis to pass. The latter way of reacting expressed the conviction that we are witnessing a reconfiguration of today’s world of working. The thoughts of these managers were to focus on opportunities to anticipate the transformational lines which will guide new ways of working and thinking about these organizations.

Bearing in mind these themes, we tried to reconstruct the routes of meaning which guided the managers’ behaviors while they were coping with the COVID-19 emergency. Altogether, three lines of development were identified, based on the managers’ shared priority and attention to the initial impact on the companies, during the 2 months when the pandemic first began.

Starting from this collective and generalized attention to contain the emergency damage, we singled out various tendencies in the development of the managers’ behaviors, which were directed in very different ways. The hypothesis is that each of these tendencies is the basis of a similar number of relationship models and expresses the different forms of reflexivity of the HR managers.”

The following discussion captures details emerging from the data analysis, summarized in Table 1.

**TABLE 1:** Different forms of reflexivity of the HR managers.

	Interview extracts	Role of human resources (employees)	Type of reflexivity	Type of agency	Time orientation
Surviving and Support	“We have set up a system of psychological support with psychologists who are assisting us with helping staff who have experienced the death of a colleague in the company to work through the grief. We introduced this psychological support system about a week ago. Obviously, I can’t talk about what people were saying, but they were telling me that the real issues at the moment are anxiety, anguish and fear.”	To comply with HR managers’ rules for coping with emergencies	Problem-oriented reflexivity	Practical evaluation	Present
Back to the past! The old reassuring models	“There was a shift of focus: first there were inspection activities, involvement-style, and then we … took action emphasizing the safety issue; we did a huge job on safety awareness. There was rethinking about the role of leaders, from controlling safety sanctions to safety services.”	To adapt themselves to the demands from top managers	Blocked reflexivity	Iteration	Past
New organizational scenarios	“Using robotics and IT in order to limit physical contact, and automation, are all trends that would have happened anyway, but which are now speeding up. As always, there are activities that disappear, and others that are on the rise.”	To adapt themselves to the role required by the models proposed	Blurred reflexivity	Iteration	Future
Crossing the Bermuda Triangle with bearings	“We trained influencers to prepare people to return to the office and help them to understand how we can help build a new way of working.”	Make a contribution to understand how to bring about a new way of organizing	Projective reflexivity	Projectivity	Future

## Results

### Surviving and Support

Analysis of the data provided a fascinating insight into the efforts of HR managers to deal with the impact that COVID-19 had on their employees. The first focus, also because of the number of citations and categories which emerged, regards a fundamental question that guided all the HR managers on how to support the women and men within their organization. Instead of various concerns regarding the hard aspects of the organization, this first category emphasizes individuals and the actions that must be activated with maximum priority in order to limit the emotional impact of COVID-19 and to preserve people’s psychological and physical health.

The supportive actions conceived by the HR chiefs regard four main topics:

1. The stress impact arising from the increased workload

Since working from home has a stronger impact in terms of time and stress than office working, it is essential to support people, firstly, by recognizing the value of this additional work and then eventually by adjusting the workload (Gozzoli et al., 2018).

“Today we are seeing a significant increase in the total workload, which is crushing us […]. We need to think about regulations to reduce the possibilities of creating alienation. Having started with 150 requests for help before the pandemic, now we are receiving 1000 requests. At the people support level, the emergency situation has meant that requests for help have multiplied. It’s an incredible situation, an enormous number of questions in need of an answer!”

2. People and time management

In this second area, the focus is on time management. It is not easy to restructure an individual’s work life and to plan daily work from home. The timing of work and the overlap of different aspects of work and personal life are a great source of stress and difficulty. One of the themes is the struggle to separate working time from the time when we usually do not work, compared with traditional organizations, work is becoming more pervasive: “I know people who start work at 6.00 in the morning. The main topic is how to organize time.”

3. Limiting employees’ fear

The third category of supportive actions regards the handling of fear, the spread of COVID-19 having generated a common dread, which leads management to take responsibility for it. Actions directed at overcoming fear usually involve specialized figures such as psychologists.

“We have set up a system of psychological support with psychologists who are assisting us with helping staff who have experienced the death of a colleague in the company to work through the grief. We introduced this psychological support system about a week ago. Obviously, I can’t talk about what people were saying, but they were telling me that the real issues at the moment are anxiety, anguish, and fear.”

This first group of managers seems to deploy an agency linked to the present and oriented toward tackling the practical problems posed by the pandemic. Emirbayer and Mische (1998) use the term “practical evaluation” to describe this type of agency, which is strongly anchored in the present and designed to find solutions that can make it possible to survive in the contingent situation. This is the first form of reflexivity which, though anchored to the imminent threat of situations, can still maintain a minimum distance to activate a form of thinking open to “problem-solving” and bringing into play all the resources which the organization has available.

### Back to the Past! the Old Reassuring Models

This cluster comprises the portion of respondents whose description of the crisis emphasizes the tragic aspect of the events which were unfolding and highlights the need to limit effects through strong actions on the part of HR. Therefore, HR was re-established as playing a central role involved in guiding people in the right direction and, at the same time, containing and limiting psychological breakdown in employees.

The key shift is that this critical situation brought about a radical change in the balance between people and the organization, a change that has the potential to produce undesirable results, such as lack of engagement, reluctance to respect burdensome new safety rules, and diverse kinds of opportunistic behavior. As such, it is only possible to overcome the crisis by establishing a strong, authoritative leadership, which people can look to as a point of reference. This is all reminiscent of certain old-fashioned management models that still exert a reassuring, seductive power. The way through the crisis involved escaping into the past and re-evoking cultural lifelines which are attractive for their apparent clarity and the central role attributed to the company’s directors. In this cluster, there was an absence of research areas and detailed study to understand which elements might be needed to cope with the crisis.

“There was a shift of focus: first there were inspection activities, involvement-style, and then we … took action emphasizing the safety issue; we did a huge job on safety awareness. There was rethinking about the role of leaders, from controlling safety sanctions to safety services.”

This is where we can place those managers who express a form of iterative agency, based on resorting to a managerial style that is obsolete, but endowed with strong emotional appeal. In this group of managers, it would seem to be impossible to put any real distance in place from the situation they are experiencing to make room for “reflexive thinking” and produce thinking of a questioning and exploratory nature. The reaction of these managers seems to be based on the irresistible attraction of emotionally reassuring models of the past, even when these are oversimplified and historically outdated (Benozzo and Colley, 2012).

### New Organizational Scenarios

In this third category, the themes raised in the interviews centered on a significant reconfiguration of work organization, starting from different data assumed as strong points of reference. What emerged was a responsive vision that focused on enhancing organization in the medium to long term. Furthermore, the objective was to adopt changes and modifications with regard to hard organizational aspects which would make it possible to adapt to new scenarios, all the while avoiding any radical processes of change.

The basic idea here is that once the critical phase is over, organizations will re-establish the “*status quo*” before COVID-19, with a few changes in ways of working, but nothing radical. The managers included in this cluster reflect on what the organizations will be like in the future and do not expect any fundamental transformations. They indicate that the crisis will have significant consequences for the financial situation of the companies, and also that if the economic recovery is slow, there will be an impact on staff numbers. Organizations will therefore be dealing with two stages (slow recovery and permanent/final recovery) connected to economic stability, which will likewise be guaranteed by downsizing companies.

The respondents’ leading hypothesis is that it will be necessary to identify innovative organizational models, which will make it possible to rethink work organization, for example in an asynchronous modality. Employees will not be allowed to work all together in the same office or workspace, smart working will be widespread, and input from the IT sector will be crucial to provide the necessary support for these new remote modes of working. In short, new organizational models will have to be found which can keep pace with organizational change. The fundamental concept of this cluster is that the main task that people will face is that of adapting. The new modes of working will require a recalibration of timing and patterns of interaction that will not always cater to individual sensitivities and needs.

“Using robotics and IT to limit physical contact, and automation, are all trends that would have happened anyway, but which are now speeding up. As always, there are activities that disappear, and others that are on the rise.”

Even though these managers employ future-oriented thinking, the question they ask themselves leaves little room for the contribution of other people in helping to design how the work organization might be changed. They pin their hopes completely on IT, which will find new modes of working thanks to technologies capable of regulating how people interact in a new way. The thinking involved in redesigning the future is delegated to IT engineers and planners. It would appear, then, that here the managers are deploying a projective agency, but one in which reflexive thinking is depleted in its creative force.

### Crossing the Bermuda Triangle With Bearings

The third pertinent area regards exploring unexpected scenarios. Assuming that the ongoing situation represents a profound change, the productive modalities and the timing of working have reached a point of no return. Work organization has its roots in production that adopt synchronous modalities and working in-person. It will be necessary to shift toward asynchronous productive models with an accompanying reduction in face-to-face people collaboration. This means that fundamental questions emerge regarding just how that legacy of relationships, upon which work organizations are historically based, can be preserved.

Trust-based relationships between people and a sense of organizational belonging are the foundations upon which companies build their competitive advantages. Acknowledging that the big question is how to identify innovative asynchronous organizational scenarios, the next challenge is how to preserve the legacy of trust between people. The concepts of this cluster regard the possibility of finding organizational devices that would allow people not to lose their central place in the work process.

By way of example, several HR directors thought about making so-called “company influencers” available, namely a coach who could support staff in the process of returning to work and help maintain involvement and commitment. The key issue was how to reflect on new conditions of cooperation to return together to a job that will be different. The main characteristic of this fourth cluster is that none of the HR managers had the answers to the questions outlined above and the only chance of finding them is to highlight some potential areas for future research paths that are emerging from the activation of people within the organization. Indeed, a contextual hypothesis could be identified so as to continue to enable them to work together.

“We trained influencers to prepare people to return to the office and help them to understand how they can help (is that right? or how to build?) a new way of working.”

Here we find a form of projective agency, and reflexivity is projected into the future. In our opinion, it is here that we find the highest form of reflexive thought capable of projecting people out of the contingent situation. The questions which guide the managers’ thoughts are strongly projected into the future, and they are linked to a potential fundamental reshaping of the way of working. They lead in turn to new questions that cannot be resolved in the short term because they need time and space in order to be addressed, and they are questions that are difficult to answer on one’s own, using pragmatic thinking. They can only be addressed by bringing into play a kind of imaginary thinking constructed together with one’s colleagues.

## Discussion

The present study interviewed a group of HR directors who responded to the impact of the COVID-19 crisis. They were all in charge of managing various workplaces, departments, and factories. Two main issues were raised during the interviews. The first is related to how managers coped with feelings of *uncertainty* in the crisis. The second regards the link between change management and reflexivity.

### Uncertainty

The role of the people in the scenarios outlined above led us to identify three types of managerial behaviors in respondents. We should point out that the first “survival and support” cluster is common to all of the respondents. All responses showed a great deal of attention to people and to the possibility of “defending” and “supporting” them using every available tool during the COVID-19 pandemic.

This study identified three types of managerial behaviors that pointed to a different conception of the processes of organizational change and the role of people. The first type of behavior occurs over a brief timespan and expresses a style of thought that is unable to accept the change taking place. The impact of the emergency generates a “freezing” of thinking and a collapse into a never-ending present, in which the only way out is to escape into a reassuring mythical past in which the re-appearance of old management models centered around the “strength” of the manager seems to provide relief (Alfes et al., 2019). The second type of behavior projects itself into the future, placing its trust in the hope that collaboration between IT and management engineers will produce new organizational models that can deal with the new emerging challenges. The third type of behavior, “Travelling with bearings” is when a person projects themselves into an uncertain future that will require a radical shift in the way we organize how people work. The uncertainty is mitigated by a series of essential points that guide HR directors:

• The challenge of adhocratic models. It is impossible to rely on abstract modeling. T-New ways of working must be identified starting from the challenges proper to each specific context.
• The challenge of asynchronous work. In the future, work will be done remotely, and this will require some profound reflection on how to rethink the relational legacy of organizations.

• The challenge of people. People will have a central role to play in identifying new sustainable working conditions. Relying on abstract models or falling under the spell of the old paradigms will not enable us to rise to the challenge of creating innovative modes of working.

### Manager Reflexivity

What emerges from this study is that HR managers reacted in very different ways to the spread of COVID-19 and these differences seem to be connected to the managers’ ability to reflect upon the situation in progress. In particular, we make use of the concept described by Cassell et al. (2019) who, emphasizing the benefits of reflective thought, points to people’s capacity for meta-thinking as being critical in enabling them to construct a complex representation of the work situation they are experiencing. This ability to face a crisis or emergency by achieving some detachment from the contingent events and expanding their reflective capacity is not evenly spread among managers. Most of our respondents exhausted their reflexive efforts either by resorting to management actions that were focused on surviving in the current situation or by using defense mechanisms that were focused on escaping to the past or projecting into a-historic future, represented by the abstract modeling of theoretical organizational models. Only a small minority of respondents (Traveling the Bermuda Triangle cluster) showed that they were able to activate reflective thought and question their strategies of problem-posing and problem-setting, projecting their managerial action into an uncertain future, in which it will be necessary to promote the involvement of all the people present in the organization to find possible forms of innovation, through widespread participation.

In our opinion, these different positions have a series of implications for how to manage human resources in times of crisis. Approaches such as “Escape to the past…” and “New organizational scenarios” which seem to be able to “tidy things up” and reassure people in a chaotic situation, may end up preventing managers from staying in touch with reality and listening to people and to their experiences, which can vary enormously.

While on the one hand the remaining approaches, “Surviving and support” and “Crossing the Bermuda Triangle,” can create uncertainty and conflicts between opposing claims, stances, and modes of experiencing and interpreting the crisis; they seem at the same time to offer managers greater opportunities to have a handle on the situation and the people involved. They can produce truly “alternative” ways of coping with the crisis, creating and strengthening new abilities to learn from experience, to be used not only in the short term but also in the medium and long term.

It seems to us, then, that this pilot study can offer initial suggestions to managers who are still involved in tackling the crisis generated by the impact of COVID-19. The first suggestion is to listen to people and to remember to bear in mind that different people react to a crisis in different ways. The second recommendation is to devote the right amount of time and space to build (together with the employees) a “framework” of meaning where colleagues can place themself, to take charge of (and make sense of) the experiences of everyone. This might mean not offering any immediate and univocal answers, nor clear indications, and might also mean listening to conflicting positions and opposing interpretations, which, if they are to be resolved, need to be heard and given some legitimacy.

### Limitations and Indication for Further Research Studies

This research was carried out over a short period, and it was possible to investigate in real-time how HR managers gave meaning to the COVID-19 emergency at the very moment in which Italy was entering lockdown. In light of these conditions, this study was able to consider only a limited sample of subjects and was therefore exploratory, and further investigation is necessary, using a bigger and wider-ranging sample of managers and companies to analyze how they responded to the crisis, the implications of reflexivity, and the social and organizational impact in the medium and long term. Furthermore, follow-up research could also monitor which management strategies are being implemented in the phases after lockdown and the consequences of these approaches.

## Data Availability Statement

The raw data supporting the conclusions of this article will be made available by the authors, without undue reservation.

## Ethics Statement

Ethical review and approval was not required for the study on human participants in accordance with the local legislation and institutional requirements. Written informed consent for participation was not required for this study in accordance with the national legislation and the institutional requirements.

## Author Contributions

All authors listed have made a substantial, direct and intellectual contribution to the work, and approved it for publication.

## Conflict of Interest

The authors declare that the research was conducted in the absence of any commercial or financial relationships that could be construed as a potential conflict of interest.

## Footnotes

1 Throughout this article, we call it “smart working” because this is the term which has been used by the press in Italy to describe this particular way of working. However, it was not the “true smart working” which involves the possibility of carrying out work activities in places other than the company headquarters or the home (for example on a train, in a park, in a library). It was not even teleworking which, to be such, requires the intervention of the company in the employee’s home in order to create a specific space where the work activity is carried out.
